# Glial fibrillary acidic protein in cerebrospinal fluid in humans is sensitive to various pre-analytical conditions: possible explanation and solution

**DOI:** 10.3389/fneur.2025.1627405

**Published:** 2025-08-01

**Authors:** Björn Evertsson, Ingela Nilsson Remahl, Max Albert Hietala, Anja Finn

**Affiliations:** ^1^Department of Clinical Neuroscience, Karolinska Institute and Department of Neurology, Karolinska University Hospital, Stockholm, Sweden; ^2^Department of Neurology, Laboratory for CSF Research, Karolinska University Hospital, Stockholm, Sweden

**Keywords:** CSF, GFAP, pre-analysis, microtube, reliability

## Abstract

Although glial fibrillary acidic protein (GFAP) has potential as a biomarker in cerebrospinal fluid, it is rarely used in clinical diagnosis due to high variability, low reliability, and poor reproducibility of results. Cerebrospinal fluid (CSF) was collected from patients (*n* = 167) at two sites at the Department of Neurology. CSF was sampled in various volumes in both 10 mL polypropylene (PP) tubes and small, filled, sealed tubes of ≤2.0 mL (microtubes) for the comparison of GFAP concentrations. The influence of pH, sample volumes during storage and transport of CSF, under different temperatures, was tested to identify the losses and increase the possibilities of replicating data for GFAP. Concentrations of GFAP were measured by a sandwich ELISA. Exposure to air, agitation, and open-close cycles increased pH and lowered CO_2_. Compared to corresponding small filled sealed tubes, routine samples stored at −20°C showed 4–30% lower concentrations of GFAP. The loss increased further at lower volumes (< 0.5 mL). A significant difference in GFAP concentrations was seen in samples taken offsite (loss 42%) and onsite (loss 24%) compared to corresponding microtubes. Concentrations of GFAP remained stable in the microtubes, at 2–8°C and at RT for up to 3 weeks. GFAP in CSF is highly sensitive to changes in pH and dependent on adequate volumes for the best results. By avoiding exposure to air and agitation, we were able to stabilize GFAP concentrations in CSF by using small, filled, sealed tubes (microtubes). This handling could have impact on other biomarkers.

## Introduction

Glial fibrillary acidic protein (GFAP) is an intermediate filament protein primarily found in astrocytes in the central nervous system (CNS). The astrocytes are essential for proper brain development, maintaining the homeostasis of ions, neurotransmitters, water, and energy and providing structural and functional support to neurons, as well as being involved in the propagation of the nerve impulse (1).

GFAP plays a crucial role in astroglia cell activation following neurodegeneration, neuroinflammation, and brain injuries, which are different types of damage to the central nervous system. GFAP is therefore an intriguing biomarker because it is rapidly released into the cerebrospinal fluid (CSF) and peripheral blood (2, 3). As a biomarker, GFAP has the potential to be used in the diagnosis of diseases in the CNS, as well as to measure the effects of medical treatment and prognosis.

The measurement of GFAP is increasingly attracting attention in a vast variety of neurological conditions (4). It is therefore of utmost importance to manage the factors affecting the final analysis. We have focused on pre-analytical potential pitfalls related to cerebrospinal fluid.

Previous studies primarily focused on biomarkers in CSF from patients with Alzheimer’s disease, which have demonstrated several clinically important variables. These include the temperature at which samples are stored, the duration nonfrozen samples can be stored, and the potential effects of additives, all of which have shown conflicting data. These studies focused on Tau, P-Tau, and amyloid, with less emphasis on GFAP, and provided a detailed demonstration of pre-analytical issues concerning biomarkers in blood (5–8).

In addition, a study from Gothenburg found that the tube material (low binding) (9) had a significant effect, and their recommendation was a unified handling protocol for CSF to minimize the pre-analytical variability (6, 10, 11).

For the specific assessment of GFAP in CSF, it was found that the concentrations decreased by 50% or more after two freeze–thaw cycles, where the authors pointed out that there may be difficulties when comparing results between collaboration centers (12).

Another discussion is the impact of the gas exchange of CO_2_ during the transportation and storage of the CSF. Since GFAP is an acidic protein with an isoelectric point of 5.8–5.7, it may be of interest to investigate how pH affects the stability of the sample during storage conditions and at various time points after sampling. It is known that pH increases rapidly in CSF due to its low non-bicarbonate buffering capacity (13). However, the pH does not increase in the same manner when a small vial (<2 mL) containing a smaller volume of CSF is used; physiological pH is preserved if the vial is filled and immediately capped (14, 15).

Several studies have been conducted to specifically measure concentrations of GFAP in CSF, and the most commonly used method is ELISA (enzyme-linked Immunosorbent assay), a sensitive immunoassay for *in vitro* quantification (pg/mL) of soluble analytes (16–19). Other methods are single molecule array (SIMOA), a powerful new technique, and electrochemiluminescence (MSD), where both orders of magnitude are more sensitive than standard ELISAs (20, 21).

To date, there is no approval for in vitro diagnostic purposes for measuring GFAP (in CSF), only for research use. On the other hand, commercially available assays are not fully validated for clinical use, especially in terms of pre-analytical conditions. Our choice of assay is an ELISA from Bertin Bioreagent (Cat No A0188, Montigny-le-Bretonneux, France). Identical protocols are also available from BioVendor (Brno, Czech Republic) and Creative Diagnostics (NY, USA).

Analytical procedures are related to the GFAP assay itself; well-known factors include operating protocols or batch-to-batch variations between kits. However, sample handling of CSF before analysis for clinical use is not yet fully defined.

To verify earlier findings that were influenced by varying pre-analytical conditions, such as freeze–thaw cycles, tube types, temperature, and volumes, we expanded the study to evaluate the impact of pH conservation. This was achieved using small tubes that were filled and sealed, with results compared across conditions and against corresponding fresh CSF samples.

The purpose of this study was to achieve the following objectives:

- define the impact of pH on the analysis of GFAP in CSF.
- evaluate the effects of additives (inhibitors) on the maintained sample quality.
- evaluate the effects of transportation of CSF between collaboration centers with respect to GFAP.
- provide suggestions for improvements to enhance sample quality prior to analysis of GFAP in human CSF.

## Methods

### Study population

Patients were recruited from the Department of Neurology at Karolinska University Hospital, located at two geographically distinct sites in Sweden: Huddinge and Solna. At the time of examination, cerebrospinal fluid (CSF) was obtained through lumbar puncture and immediately divided into polypropylene tubes according to the study protocol.

### Study protocol

#### Part 1: Stability test: freeze–thaw cycles

To confirm previous findings and to compare with concentrations of GFAP in the filled and sealed small tube (microtube). CSF from two individuals was equally aliquoted into new polypropylene 3.5 mL tubes (Sarstedt, Cat No 555.535) and stored at −20°C for 1 week. During this time, these samples underwent repeated freeze–thaw cycles (5x) to room temperature (RT) pending analysis.

#### Part 2: Storage at different volumes, tubes, temperatures, and pH levels

The first sample of CSF was discarded, and the next one was designated as the origin tube (Sarstedt, Cat No: 62.9924.284) from which up to 10 mL of CSF was collected. The following tubes were microtubes (Cryotubes, 1.5 mL or 2.0 mL), numbered consecutively. CSF was dropped into these tubes, which were immediately sealed once filled to prevent further exposure to air. The CSF was centrifuged for 10 min at 2000 g at RT to remove any cells and debris, thereafter aliquoted in separate volumes (0.15 mL to 2 mL) into polypropylene 3.5 mL tubes and stored at RT, refrigerator 2–8°C, and freezer −20°C. The microtubes were stored at RT and/or 2–8°C, and one aliquot from the origin sample was stored at −20°C. pH and CO_2_ were checked in the origin tubes stored at RT as well as in the corresponding microtubes to verify the loss of CO_2_ using a Radiometer ABL 800 Flex, blood gas analyzer.

#### Part 3: Addition of protease and phosphatase inhibitor

CSF was collected from three individuals and immediately divided into eight polypropylene tubes, each in equal amounts. Protease and phosphatase inhibitor (Cat # ab 201,120, Abcam, Cambridge, UK) was added to a final dilution of 1:9 (270 μL sample + 30 μL inhibitor cocktail) into four tubes from each patient. Samples were stored at RT, refrigerator 2–8°C, freezer −20°C, and at −80°C pending analysis.

#### Part 4: Transportation, long-term preservation, origin, compared to the microtube

At the time of medical examination, CSF was obtained through lumbar puncture directly into polypropylene tubes (up to 3 mL and microtubes of 2.0 mL) for the measurement of GFAP. Samples were sent to the laboratory from two sites. Off-site, *n* = 75, on-site, *n* = 74. For the evaluation of transport, aliquots of CSF were stored at −20°C (routine) and 2–8°C (microtube) and analyzed continuously upon arrival in the laboratory. For the long-term preservation test, the samples were analyzed on two occasions, 3 weeks apart.

To confirm the previous findings, an additional study (*n* = 9) was conducted to investigate the loss of GFAP during transportation. The CSF was collected in an origin tube and four microtubes. From the origin tube, which contained 6 mL of CSF, two 2 mL portions were aliquoted into separate 10 mL tubes and kept on-site. The remaining 2 mL, in the origin tube, and on microtube was transported off-site and back. under ambient conditions, while corresponding samples were kept on-site at room temperature and 2–8°C.

### Principle of the GFAP assay

The measurement of glial fibrillary protein (GFAP) in human CSF is based on a sandwich enzyme immunoassay technique. We used a commercially available ELISA KIT (Cat# A05188, Bertin Pharm, Montigny-le-Bretonneux, France) where an antibody specific for human GFAP was pre-coated onto a microplate. The standard curve was set to 0–5,000 ng/L and not further diluted. Samples were added to the plate, in duplicate, after a 1:3 dilution in ELISA buffer and then incubated for 2 h. The following steps were performed according to the manufacturer’s protocol. Plates were read at 450 nm using a plate reader (SpektraMax 190, Molecular Devices, UK), and the results were back-calculated according to the corresponding standards. Results were presented as means of the duplicates multiplied by the dilution factor.

The GFAP KIT was tested for quality control and consistency of standard curves across the plates. For plate-to-plate variability, the coefficient of variation (CV) was assessed using the provided quality controls at two concentrations, showing 9.6 and 11.3%, respectively. The limit of detection (LOD) was set by the manufacturer to 45 ng/L, and the limit of quantification (LOQ) at the present laboratory was 62.5 ng/L.

Statistical analysis: Parametric tests were used to compare groups, including a t-test for two-group comparisons and one-way ANOVA, corrected with Dunnett’s test, for multiple comparisons. All statistical analyses were performed, and figures were computed in GraphPad Prism 8.

## Results

### Part 1

As previously shown, when samples are thawed and refrozen, the GFAP values gradually decline or become undetectable, depending on the starting value. Fresh sample 1 (=origin) 926 ng/L, small sealed tube 1,188 ng/L, and after five thawing, 435 ng/L. Fresh sample 2 (=origin), 210 ng/L, small sealed tube 477 ng/L, and after three thawing, not detectable.

### Part 2

Comparison of GFAP concentrations between different volumes in CSF, ranging from 150 μL to a routine standard of 500 μL and up to 2.0 mL of CSF, frozen at −20°C, showed that the value of GFAP is affected depending on pre-handling of the CSF samples. The worst-case scenario was observed with low volumes (< 0.5 mL) at concentrations less than 1,055 ng/L, stored at −20°C, resulting in a loss of GFAP of up to 41%. The volume collected in 10 mL PP tubes is a factor that preserves GFAP in CSF, not only the temperature. Larger volumes (up to 2 mL) of CSF and higher concentrations of GFAP yielded more reliable results. Values were compared to those from a microtube kept at 2–8°C until analysis, and concentrations of GFAP in the routine standard volume showed between 74 and 96% of the concentrations in the microtube (*n* = 5) (see Supplementary Table).

pH in CSF was also shown to be affected by various aspects of pre-analytical handling of the sample. A lower volume (not filled tubes) of CSF, as well as agitation of the sample, increased the pH and lowered the CO_2_ (Table 1).

**Table 1:** Changes in pH value and CO_2_ in CSF, depending on the pre-analytical handling.

Sample ID (CSF)	Comment	pH	CO_2_
S04	not filled, agitated	>8	3.03
S09	transferred to microtube, agitated	>8	3.02
S05-2	reanalysed, agitated	8	3.81
S01-2	reanalysed, second opening	7.56	4.14
S02-2	reanalysed, second opening	7.53	3.86
S08	not filled	7.52	4.46
S02-1	first opening	7.42	4.23
S06	first opening	7.47	4.56
S07	first opening	7.48	5.01
S05-1	first opening	7.48	n.d.
S03	first opening	7.49	4.66
S01-1	first opening	7.51	4.48

### Part 3

Experiments evaluating the effects of preservatives were carried out using an inhibitor (protease and phosphatase inhibitor cocktail) and comparing that to the use of phosphate-buffered saline (PBS) or Milli-Q H_2_O at any temperature. No significant difference was observed compared to the addition of water or PBS (no graphs shown).

### Part 4

**Figure 1:**
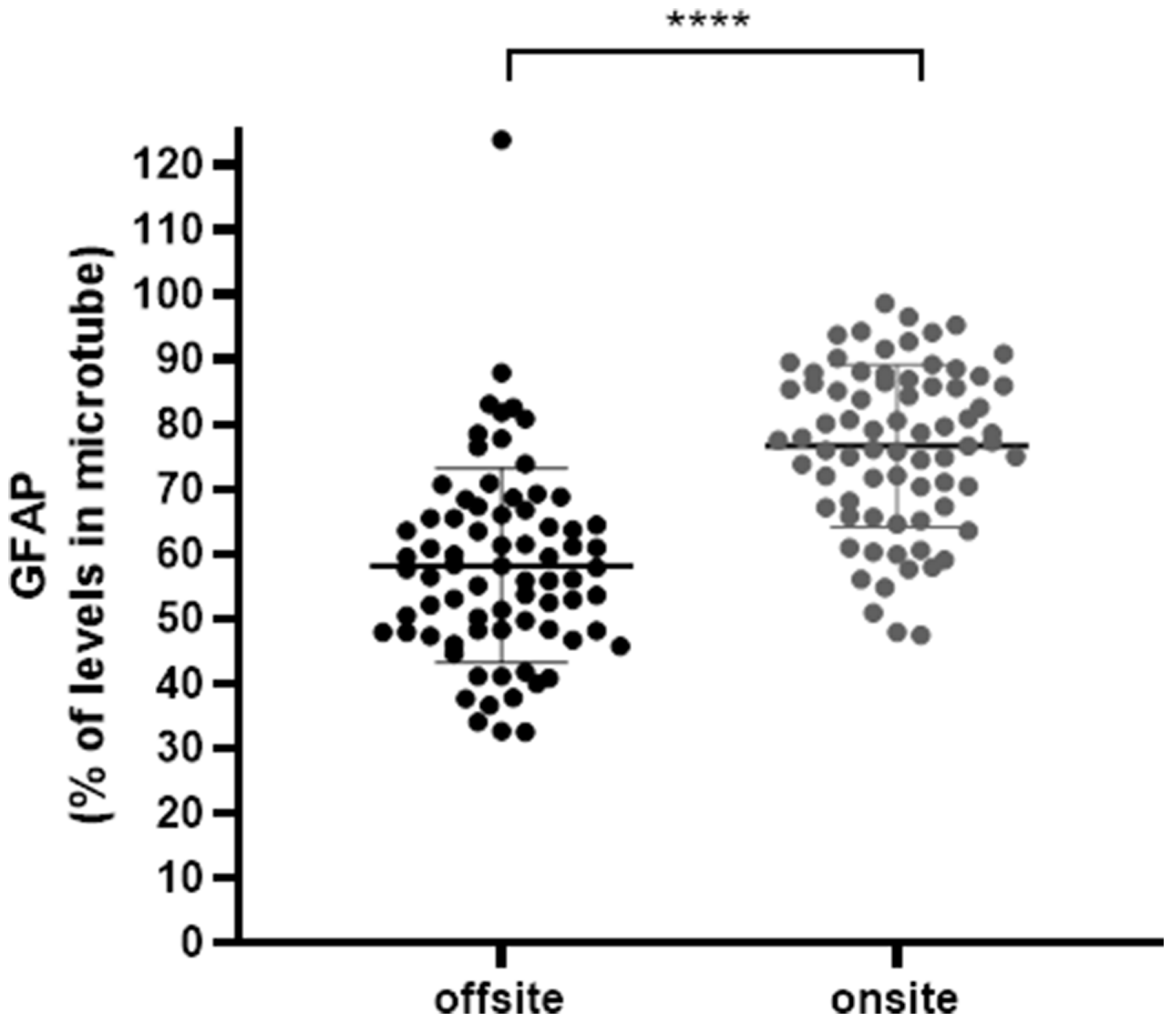
GFAP concentrations in samples from on-site and off-site compared to their corresponding concentrations in the small filled sealed tubes (microtube), as a percentage. The graph compares concentrations of GFAP in 500 μL of CSF that was immediately frozen at −20°C and thawed for analysis, with the values of GFAP in microtubes. Samples collected off-site were in transit for up to 48 h, under ambient conditions, from a spinal tap; off-site *n* = 75, on-site *n* = 74. Mean SD. **** *p* ≤ 0.0001.

To investigate the effect of sample storage during transportation on GFAP concentrations measured in small filled sealed tubes (microtubes) and origin 10 mL PP tubes, we included 74 patients on-site and 75 patients off-site. The results showed that transportation had a greater impact on the GFAP concentrations measured in the origin 10 mL PP tubes than in microtubes. There was a significant difference in the GFAP losses between samples collected on-site and off-site, compared to their corresponding microtubes (Figure 1). The *p*-value **** = *p* ≤ 0^.^0001. The microtube was found to preserve GFAP in CSF better when transporting samples.

**Figure 2:**
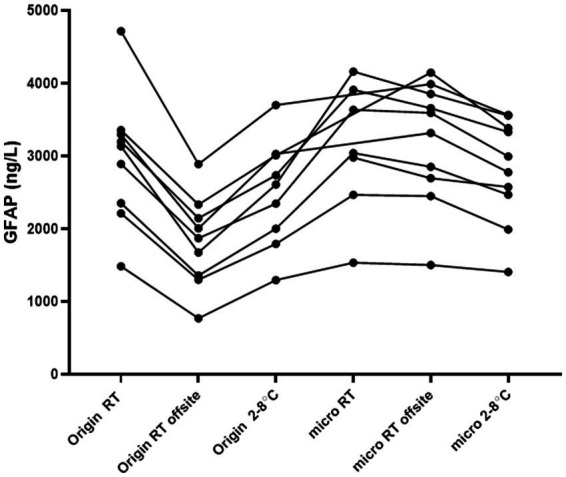
Concentrations of GFAP in CSF in various tube types and pre-analytical conditions. Off-site samples were in transit, back and forth, up to 48 h under ambient conditions. On-site samples were kept on the bench or at 2–8°C. Each line represents samples from one individual that have been handled in various conditions. Origin = routine sample in a 10 mL PP tube, micro: microtube, or a 2 mL filled PP tube, and RT: room temperature.

A small study (*n* = 9) was conducted to verify the findings mentioned above. The study aimed to determine the differences in samples taken from the same individual when sent back and forth off-site, and compare them with corresponding samples kept on-site at room temperature and 2–8°C. In summary, the 2 mL CSF origin tube sent off-site showed a notable decrease in GFAP concentrations when compared to all other tested tubes and conditions. Conversely, GFAP concentrations remained stable in all microtubes kept at room temperature (Figure 2).

**Figure 3:**
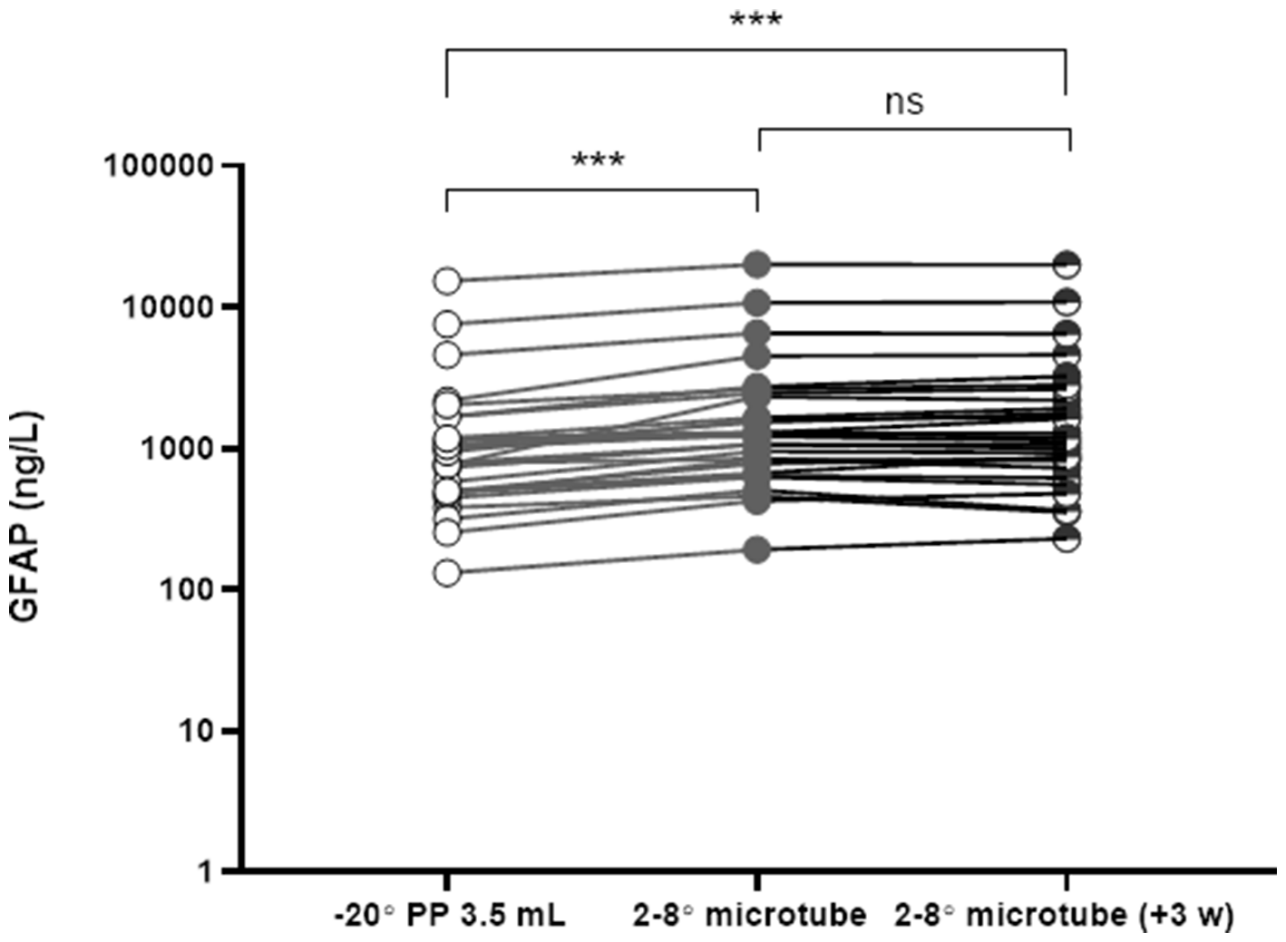
GFAP concentrations in CSF: comparison of origin tubes and microtubes over time. CSF was collected simultaneously in 10 mL polypropylene (PP) tubes and microtubes. A 500 μL aliquot was transferred into 3.5 mL PP tubes, frozen at −20°C, and analyzed as a routine sample. Microtubes were stored at 2–8°C and analyzed twice: once within 1 week of lumbar puncture and again after 3 weeks. *n* = 16. ns = non-significant; ***p ≤ 0.001.

For the long-term preservation study (*n* = 16), CSF was collected in a 10 mL PP tube and microtubes from the patients at the same time. Microtubes were kept in 2–8°C throughout the study. 500 μL of CSF from a 10 mL PP tube was frozen at −20°C and analyzed once after thawing; the corresponding microtube was analyzed twice, once within a week of spinal tap and once again after 3 weeks. All samples were taken on-site. Higher concentrations of GFAP were consistently analyzed from microtubes compared to those in 10 mL PP tubes. The microtubes were also found to keep the concentrations of GFAP stable in 2–8°C for up to 3 weeks (Figure 3). There was a significant difference in GFAP concentrations when comparing 10 mL PP tubes to that of GFAP in microtubes *** = *p* ≤ 0.001.

#### Interpretation of results

##### Possible explanation

Agitation and air exposure can lead to CO₂ loss, resulting in an increased pH. Moreover, low CSF volumes and protein adsorption to the tube walls may contribute to reduced GFAP concentrations.

##### Proposed solution

Using a small, filled, and sealed tube at the time of collection minimizes air exposure, maintains adequate sample volume, reduces adsorption to tube surfaces, and helps preserve CO₂ and pH, ultimately improving GFAP stability.

## Discussion

Glial fibrillary acidic protein in CSF serves as an important diagnostic biomarker. However, its clinical utility is limited by significant variability and challenges in replicating findings. As demonstrated in previous research (12) and corroborated by our study, GFAP concentrations are sensitive to pre-analytical conditions such as freeze–thaw cycles, (8, 22) temperature fluctuations, and storage practices, i.e., the impact of storage volume of CSF (23). The main recommendation to avoid these issues is the use of fresh samples.

To address these challenges, our study explored potential solutions to enhance the stability of GFAP in CSF.

In the initial test, we aimed to measure pH and CO_2_ concentrations in CSF. We first conducted these measurements in a sealed system and then exposed the samples to various pre-analytical conditions, such as oxygen exposure, movement, and multiple open-close cycles. Through this process, we observed an increase in pH and a loss of CO_2_.

We did not specifically focus on the type of tube used (cryotube or microtube). Instead, we concentrated on the properties these tubes provide, such as preventing air exposure (which helps maintain pH), tolerating movement, and offering a defined volume. All these factors are important for preserving GFAP concentrations in the CSF.

We used small cryotubes, referred to as microtubes, compared to standard CSF collection tubes (origin, fresh sample) throughout the entire study for measuring GFAP in CSF.

Firstly, our findings also revealed that increasing CSF volume approaches the GFAP concentrations observed in the filled, sealed tubes, stored at 2–8°C. This provides a practical strategy for reducing pre-analytical variability. However, the acquisition of large CSF volumes can be challenging in clinical settings, making microtubes an appealing alternative for collecting smaller volumes while preserving biomarker stability. Notably, our research highlighted that larger volumes only confer benefits when samples are frozen before transport and not subjected to multiple freeze–thaw cycles (Supplementary Table).

We demonstrated that GFAP remains stable in these small, filled tubes, stored at 2–8°C for over 21 days, illustrating their utility for non-frozen storage and transportation between sites. These findings suggest that microtubes could serve as a practical alternative to freezing in clinical practice, though their suitability diminishes when analyzing large batches or requiring long-term storage. Nevertheless, microtubes outperformed other methods in maintaining stable GFAP values, even at room temperature, though we were unable to assess their long-term stability under such conditions. Bacterial growth remains a potential concern for storing at room temperature.

In evaluating the impact of transportation and site-specific factors, we observed significant variability within the cohort. We noted a substantial percentage loss of GFAP concentrations in off-site samples compared to on-site collections. These observations underscore the influence of transport and handling time on GFAP stability, further highlighting the utility of sealed, filled microtubes in mitigating these effects.

We propose two primary mechanisms for the superior performance of microtubes in preserving GFAP. First, increased pH due to CO2 gas exchange in opened tubes may alter the protein’s conformation, reducing its detectability by ELISA. This pH shift is minimized in filled sealed tubes, preserving GFAP stability. Second, the surface area-to-volume ratio in larger tubes may lead to greater adsorption of GFAP onto tube walls, reducing its measurable concentrations. Our study demonstrates that a fixed sample volume, when combined with partial filling of larger tubes, results in variations in measured concentrations. In summary, the findings suggest that complete filling of tubes reduces the surface area-to-volume ratio, even at low sample volumes, which may in turn limit analyte loss due to adsorption. These mechanisms likely act synergistically, emphasizing the importance of sealed systems and minimal CSF exposure during handling. This finding is discussed in detail for other proteins in CSF related to Alzheimer’s disease (23).

Our findings underscore the advantages of microtubes for collecting and storing CSF biomarkers, particularly GFAP, which is sensitive to pre-analytical errors. Implementing this approach in clinical settings could improve the reliability of CSF biomarker analyses, minimizing risks such as falsely low concentrations and artifact-related measurements. Despite the technical challenges of handling small tubes, their ability to preserve biomarker stability supports their clinical utility.

While our study focused on GFAP, the implications of these findings likely extend to other biomarkers sensitive to pre-analytical handling. Addressing these errors could significantly impact the perceived reliability of biomarkers in CSF analysis.

Future research should explore the application of microtubes to additional biomarkers and their broader impact on clinical practice.

Interestingly, a previous study found that our data indicated no differences in neurofilament light (NFL) concentrations between microtubes and standard tubes, suggesting that not all biomarkers are equally affected by pre-analytical conditions. This highlights the need for biomarker-specific handling protocols, as exemplified by the differences in clinical adoption between NFL and GFAP.

GFAP, though a more recent addition to clinical practice, is gaining recognition for its diagnostic utility in conditions such as neuromyelitis optica (NMO) and other CNS disorders (24–30). All demonstrate that GFAP in CSF has a dynamic range in the mentioned diseases, while reference values are rarely presented (31, 32).

Finally, blood is not affected in the same manner (5, 8, 33, 34). Blood is more easily obtained (volume is not an issue), and it has several different buffering systems that stabilize pH besides bicarbonate.

Our study did not evaluate the impact of tube materials, though differences in materials, such as polystyrene versus polypropylene, may influence biomarker stability. Polypropylene remains the standard for CSF analyses (35), offering a suitable baseline for future investigations.

To conclude, the present study demonstrated that GFAP in CSF is highly sensitive to pre-analytical handling conditions, including exposure to air, agitation, tube type, sample volume, and transportation protocols. Sealed, filled microtubes stored at 2–8°C maintained consistent GFAP concentrations for up to 3 weeks, providing a reproducible and reliable alternative to standard CSF handling. These findings suggest that microtubes are superior for preserving GFAP and potentially other biomarkers, underscoring the importance of optimized pre-analytical protocols in clinical practice. Our study suggests the usage of a small, filled tube, along with adjustments of reference values.

## Data Availability

The original contributions presented in the study are included in the article/Supplementary material, further inquiries can be directed to the corresponding author.
