# Supplementary material for: Glial fibrillary acidic protein in cerebrospinal fluid in humans is sensitive to various pre-analytical conditions: possible explanation and solution
Source: Front Neurol. 2025 Aug 1;16:1627405. doi: 10.3389/fneur.2025.1627405 (PMC12354587; doi:10.3389/fneur.2025.1627405)
Supplement: Supplementary file 1 [file Table_1.docx]

Appendix 1. Comparison of GFAP concentrations across different CSF volumes, tube sizes, and storage conditions.

|  |  |  | Individuals | | | | |  |
| --- | --- | --- | --- | --- | --- | --- | --- | --- |
|  | Condition | Volume (mL) | 1 | 2 | 3 | 4 | 5 | Mean |
| Suggestion | **+4°C** microtube ng/L | 2.0 | 686 | 914 | 915 | 1055 | 1386 | 991 |
| Routine | **-20°C** (3.5mL PP tube) ng/L | 0.5 | 510 | 674 | 858 | 742 | 1335 | 824 |
|  | **% of levels in microtube** |  | 74 | 74 | 94 | 70 | 96 | 82 |
|  |  |  |  |  |  |  |  |  |
|  |  | volume | **% of levels in microtube** | | | | | mean (%) |
|  | **-20°C** (3.5mL tube) | 0.15 | 67 | 64 | 64 | 59 | 84 | 68 |
|  | **-20°C** (3.5mL tube) | 0.25 | 71 | 58 | 83 | 70 | 96 | 76 |
|  | **-20°C** (3.5mL tube) | 1.0 | 85 | 79 | 95 | 83 | 96 | 88 |
|  | **-20°C** (3.5mL tube) | 2.0 | 85 | 75 | 99 | 99 | 114 | 94 |
|  | **+4°C** (10mL PP tube) | 2.0 | 84 | 79 | 85 | 83 | 100 | 86 |
|  | **-20°C** (10mL PP tube) | 2.0 | 72 | 78 | 97 | 76 | 106 | 86 |

Concentrations are compared, as percentages, to a microtube (set at 100%) stored at 2–8 °C until analysis.

The routine sample is collected in a 10 mL polypropylene (PP) tube, from which 500 µL is transferred into a 3.5 mL PP tube and stored at −20 °C.

Increasing CSF volume leads to GFAP concentrations approaching those observed in microtubes stored at 2–8 °C.
